# Protein secretion in *Lactococcus lactis *: an efficient way to increase the overall heterologous protein production

**DOI:** 10.1186/1475-2859-4-2

**Published:** 2005-01-04

**Authors:** Yves Le Loir, Vasco Azevedo, Sergio C Oliveira, Daniela A Freitas, Anderson Miyoshi, Luis G Bermúdez-Humarán, Sébastien Nouaille, Luciana A Ribeiro, Sophie Leclercq, Jane E Gabriel, Valeria D Guimaraes, Maricê N Oliveira, Cathy Charlier, Michel Gautier, Philippe Langella

**Affiliations:** 1 Laboratoire de Microbiologie UMR1253 STLO, INRA-Agrocampus, 65, rue de Saint Brieuc CS84215, 35042 Rennes cedex, France; 2 Institute of Biological Sciences, Federal University of Minas Geiras (ICB-UFMG), Belo Horizonte-MG, Brazil; 3 Unité de Recherches Laitières et de Génétique Appliquée, Institut National de la Recherche Agronomique, Domaine de Vilvert, 78352 Jouy en Josas Cedex, France

## Abstract

*Lactococcus lactis*, the model lactic acid bacterium (LAB), is a food grade and well-characterized Gram positive bacterium. It is a good candidate for heterologous protein delivery in foodstuff or in the digestive tract. *L. lactis *can also be used as a protein producer in fermentor. Many heterologous proteins have already been produced in *L. lactis *but only few reports allow comparing production yields for a given protein either produced intracellularly or secreted in the medium. Here, we review several works evaluating the influence of the localization on the production yields of several heterologous proteins produced in *L. lactis*. The questions of size limits, conformation, and proteolysis are addressed and discussed with regard to protein yields. These data show that i) secretion is preferable to cytoplasmic production; ii) secretion enhancement (by signal peptide and propeptide optimization) results in increased production yield; iii) protein conformation rather than protein size can impair secretion and thus alter production yields; and iv) fusion of a stable protein can stabilize labile proteins. The role of intracellular proteolysis on heterologous cytoplasmic proteins and precursors is discussed. The new challenges now are the development of food grade systems and the identification and optimization of host factors affecting heterologous protein production not only in *L. lactis*, but also in other LAB species.

## Introduction

Lactic Acid Bacteria (LAB) are anaerobic Gram positive bacteria with a GRAS (Generally Regarded As Safe) status. They are also food grade bacteria, and therefore, they can be used for the delivery of proteins of interest in foodstuff or in the digestive tract. A last advantage compared to other well-known protein producers is that *L. lactis *does not produce LPS or any proteases as *Escherichia coli *or *Bacillus subtilis *do, respectively.

In the last two decades, genetic tools for the model LAB, *Lactococcus lactis*, were developed: transformation protocols, cloning- or screening-vectors [1,2], and mutagenesis systems [3] are now available. Moreover *L. lactis *genome is entirely sequenced [4]. Many protein expression- and targeting-systems have also been designed for *L. lactis *[5-7]. These systems have been used to engineer *L. lactis *for the intra- or extra-cellular production of numerous proteins of viral, bacterial or eukaryotic origins (Table 1). To produce a protein of interest in fermentors, secretion is generally preferred to cytoplasmic production because it allows continuous culture and simplifies purification. To use *L. lactis *as a protein delivery vehicle in the digestive tract of humans or animals, secretion is also preferable because it facilitates interaction between the protein (e.g. enzyme or antigen) and its target (substrate or immune system).

**Table 1:** Heterologous proteins produced in *Lactococcus lactis*.

**Proteins**	**Gene**	**Origin**	**Location**	**References**
**Reporter**				

Nuc	*nuc*	*Staphylococcus aureus*	Cytoplasmic / secreted / anchored	[6, 16]
β-lactamase	*bla*	*Escherichia coli*	secreted	[44]
β-galactosidase	*β*-*gal*	*Clostridium acetobutylicum*	cytoplasmic	[45]
lactamase	*lacL*, *lacM*	*Leuconostoc mesenteroides*	cytoplasmic	[46]
α-amylase	*amyS*	*Geobacillus *(*formerly Bacillus*) *stearothermophilus*	secreted	[47] [18]
α-amylase	*amyL*	*Bacillus licheniformis*	secreted	[48]
Chloramphenicol Acetyl Transferase	*cat*-*86*	*Bacillus pumilus*	cytoplasmic	[49]
M6		*Streptococcus pyogenes*	anchored	[12]
Green fluorescent protein	*gfp*	*Aequoria victoria *(jellyfish)	cytoplasmic	[50]
luciferase	*luxAB*	*Vibrio harveyi*	cytoplasmic	[51]
luciferase	*Vf lux*	*Vibrio fischeri*	cytoplasmic	[52]
Streptavidin	*SA*	*Streptomyces avidinii*	anchored	[11]
β-glucuronidase	*gus*	*Escherichia coli*	cytoplasmic	[53]
**Bacterial antigens**				

L7/L12	L7/L12	*Brucella abortus*	Cytoplasmic/secreted/anchored	[19]
Urease subunit B		*Helicobacter pilori*	secreted	[54]
TTFC	ttfc	*Clostridium tetani*	secreted	[55]
**Eukaryotic antigen**				

GLURP-MSP3 fusion protein		*Plasmodium falciparum*	secreted	[56]
**Viral antigens**				

E7	E7	HPV type-16	cytoplasmic/secreted/anchored	[20] [57]
NSP4	NSP4	Bovine coronavirus	cytoplasmic	[29]
BCV epitope	BCV	Bovine coronavirus	secreted	[58]
VP8 subunit of VP4	VP8*	rotavirus	secreted	[59]
**Interleukins**				

IL-2	IL-2	Mouse	secreted	[60]
IL-6	IL-6	Mouse	secreted	[61]
IL-10	IL-10	Mouse	secreted	[21]
IL-12	IL-12	Mouse	Secreted	[22]
IFN-ω	IFN-ω	Ovine	secreted	[5]
**Allergens**				

BLG	Blg	Bovine	cytoplasmic/secreted	[13, 30, 36]
	Epitope Blg41–60	Bovine	secreted	
**Virulence factors**				

Fibronectin binding protein A	*fnbpA*	*Staphylococcus aureus*	anchored	[62]
Clumping factor A	*clfA*	*Staphylococcus aureus*	anchored	[63]
Clumping factor A and B	*clfB*	*Staphylococcus aureus*	anchored	[64]
serine-aspartate repeat protein	*sdrE*	*Staphylococcus aureus*	anchored	[64]
Protein A	*spA*	*Staphylococcus aureus*	anchored	[11]
Enterotoxin A	*sea*	*Staphylococcus aureus*	secreted	C. Charlier^(a)^ Unpublished results
Aggregation substance	*asc10*	*Enterococcus faecalis*	anchored	[65]
Capsular polysaccharides	*cps *genes	*Streptococcus pneumoniae*	CPS excreted	[66]
Internalin	*inlA*	*Listeria monocytogenes*	anchored	V. Guimarães^(b)^ Unpublished results
**Bacteriocins**				

ABP-118	*abp118*	*Lactobacillus salivarius subsp. salivarius*	secreted	[67]
Enterocin A	*ent *genes	*Enterococcus faecium*	secreted	[68]
Pediocin PA-1	*ped *genes	*Pediococcus acidilactici*	secreted	[68]
colicin V		*Escherichia coli*	secreted	[69]
**Enzymes**				

heat-stable alpha-glucosidase	*malA*	*Sulfolobus solfataricus*	cytoplasmic	[70]
Bacteriophage lytic enzyme	*ply 118*	*Listeria monocytogenes *bacteriophage	secreted	[71]
lysozyme	*hel*	Hen egg white	cytoplasmic	[72]
Neutral protease	*npr*	Bacillus subtilis	secreted	[73]
Aminopeptidase N	*pepN*	*Lactobacillus helveticus*	secreted	[74]
Cell Surface Protease	*prtB*	*Lactobacillus delbrueckii subsp. bulgaricus*	anchored	[13]
Dextrane sucrase	*dsrD*	*Leuconostoc mesenteroides*	secreted	[28]
Streptodornase	*sdc*	*Streptococcus equisimilis*	secreted	[75]
prochymosin	*PC*	Bovine	secreted	[76]
lipase	*lip*	*Staphylococcus hyicus*	secreted	[77]
plasmin		Bovine	secreted	[78]
**others**				

F18 fimbrial adhesin (receptor binding domain)	*fedF*	*Escherichia coli*	Secreted / anchored	[27]
S-layer protein	*slpH*	*Lactobacillus helveticus*	cell wall associated	[79]

^(a) ^: Laboratoire de Microbiologie UMR1253 INRA Agrocampus, 65 rue de Saint Brieuc CS84215, 35042 Rennes cedex

^(b) ^: Unité de Recherches Laitières et de Génétique Appliquée, Institut National de la Recherche Agronomique, Domaine de Vilvert, 78352 Jouy en Josas Cedex, France

In LAB, like in other Gram positive bacteria, secreted proteins are synthesized as a precursor containing an N-terminal extension called the signal peptide (SP) and the mature moiety of the protein. Precursors are recognized by the host secretion machinery and translocated across the cytoplasmic membrane (early steps). The SP is then cleaved and degraded, and the mature protein is released in the culture supernatant (late steps). Sometimes, secreted proteins require subsequent folding and maturation steps to acquire their active conformation [8].

In most of the works describing heterologous protein production by recombinant lactococci, only one cellular-location (i.e. cytoplasm, external media or surface anchored) is described. Only a few works report the production of a given protein in different locations using the same backbone vector, the same induction level and or promoter strength, allowing thus a rigorous comparison of the production yields of cytoplasmic and secreted forms.

Here, six examples of different heterologous proteins produced in *L. lactis *in both secreted and cytoplasmic forms are reviewed and discussed. Our major conclusion is that the best production yields are observed in most of these cases with secretion (up to five-fold higher than with cytoplasmic production). Moreover, engineering the expression cassette to enhance the secretion efficiency (SE, proportion of the total protein detected as mature form in the supernatant) resulted in increased overall amounts of the protein. *L. lactis *is able to secrete proteins ranging from low-(< 10 kDa) to high-(> 160 kDa) molecular mass through a Sec-dependant pathway. Altogether, these observations suggest that i) heterologous proteins produced in *L. lactis *are prone to intracellular degradation whereas secretion allows the precursor to escape proteolysis, and ii) conformation rather than protein size is the predominant feature that can impair SE. New perspectives are now opened in the studies of heterologous protein production in *L. lactis*. Indeed, there is a need for food grade systems and for a better understanding of the host factors influencing heterologous protein secretion in *L. lactis *. For example, HtrA-mediated proteolysis (HtrA is the unique housekeeping protease at the cell surface) is now well-characterized in *L. lactis *[9] and can be overcome by use of a *htrA L. lactis *strain designed for stable heterologous protein secretion [10]. However, intracellular proteolysis (involving Clp complex -the major cytoplasmic housekeeping protease-, and probably other cellular components) remains poorly understood and is also discussed here.

## Get out to get more

**Figure 1:**
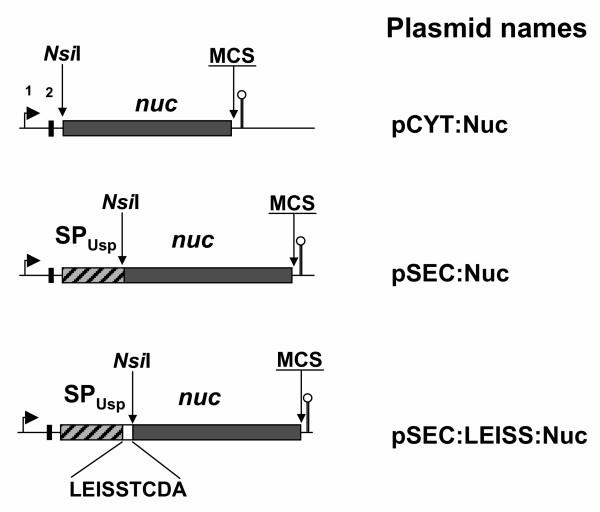
**Schematic representation of Nuc cassettes for controlled and targeted production in *L. lactis. ***For details about plasmid constructions and contents see Bermúdez-Humarán et al. (2003) [5]. Plasmid backbone is a derivative of the rolling circle plasmid pWVO1, an *E. coli*-Gram positive shuttle vector. Arrows (1) indicate the presence of the nisin-inducible promoter (P_*nisA*_); solid vertical bars (2) indicate the Ribosome Binding Site of the *usp45 *gene; the striped bar indicate signal peptide of the *usp45 *gene (SP_Usp_); the white bar indicates the insertion of LEISSTCDA synthetic propeptide [18]; dark gray bars indicates Nuc mature coding sequence; stem-loop structures indicate *trpA *transcription terminators (not to scale). A *Nsi*I restriction site comprises the ATG start codon (in pCYT) or the last two residues of SP_Usp _(pSEC) and allows a simple and one-step cloning of the cassettes corresponding to the mature proteins for cytoplasmic production (pCYT) or secretion (pSEC).

Genetic tools to target a given protein in different cellular compartments were developed using several reporter proteins [6,11-13] (Table 1). The staphylococcal nuclease (Nuc) is a well-characterized secreted protein whose activity is readily detectable by petri plate assay and it has been used as a reporter protein for secretion studies in several Gram positive hosts [14-16]. In *L. lactis*, Nuc was used to develop protein targeting- [6] and SP screening-systems [1,2]. Nuc was chosen to develop the pCYT and pSEC vectors for controlled production in *L. lactis *of cytoplasmic or secreted forms of a protein of interest, respectively (Fig. 1) [5]. The pCYT and pSEC plasmids, where expression is controlled by a nisin inducible promoter, should be used in *L. lactis *NZ9000 (hereafter referred to as NZ) strain bearing a *nisR*,*K *chromosomal cassette, required for the nisin signal transduction [17]. In each case described below, protein sample concentration was adjusted to the cell density of the producing culture (for details see [18]). At similar induction levels in lactococcal strains containing pCYT:Nuc and pSEC:Nuc vectors, the highest production yields were observed with the secreted Nuc form (Table 2). Similar results were obtained with constitutive *nuc *expression cassettes for cytoplasmic and secreted forms. Nuc was the first heterologous protein where highest protein yields were obtained with the secreted form.

**Table 2:** Comparison of the protein yields in secreted vs cytoplasmic production.

**Protein**	**Quantification of the secreted form^1^**	**Quantification of the cytoplasmic form^1^**	**Ratio sec/cyto**	**References**
Nuc	20 mg/L	3 mg/L	6	[5]
L7/L12	3 mg/L	0.5 mg/L	6	[19]
E7 (expo)*	nd	nd	2 to 3	[20]
E7 (stat)*	nd	nd	> 10	[20]
IFN-ω	309 mg/L	159 mg/L	2	[5]

1: protein samples were adjusted to the cell density and protein quantification was performed as described in the references either by western blot or by ELISA.

*: E7 was not quantified but ratio was calculated by scanning the western blot signals and comparing their intensity as described in the corresponding reference.

nd: not determined

Similar results were obtained for the production of a *Brucella abortus *ribosomal protein. *B. abortus *is a facultative intracellular Gram negative bacterial pathogen that infects human and animals by entry through the digestive tract. The immunogenic *B. abortus *ribosomal protein L7/L12 is a promising candidate for the development of oral live vaccines against brucellosis using *L. lactis *as a delivery vector. L7/L12 was produced in *L. lactis *using pCYT and pSEC vectors [19]. Similarly to Nuc production, the production yield of secreted L7/L12 was reproducibly and significantly higher than that of the cytoplasmic form (Table 2).

Another example of higher protein yields in secreted vs cytoplasmic form is the production the human papillomavirus type 16 (HPV-16) E7 antigen, a good candidate for the development of therapeutic vaccines against HPV-16 induced cervical cancer. The E7 protein is constitutively produced in cervical carcinomas and interacts with several cell compounds. E7 was produced in a cytoplasmic and a secreted form in *L. lactis *[20]. Using similar induction level in exponential phase cultures, E7 production was higher for the secreted form than for the cytoplasmic form (Table 2). This difference was even higher when induction occurred in late-exponential phase, where intracellular E7 was detected at only trace amount whereas secreted E7 was accumulated in NZ(pSEC:E7) culture supernatant (see below). Thus, production of E7 clearly illustrates the fact that secretion results in higher yields in *L. lactis*.

Production of ovine interferon omega (IFN-ω) further illustrates this observation. In the case of poorly immunogenic antigens, co-delivery of an immuno-stimulator protein can enhance the immune response of the host. In order to optimize the use of lactococci as live vaccines, the production of cytokines was investigated in *L. lactis *[5,21,22]. IFN-ω is a cytokine able to confer resistance to enteric viruses in the digestive tract by reduction of viral penetration and by inhibition of intracellular multiplication of the viruses. Delivery of ovine IFN-ω in the digestive tract by recombinant *L. lactis *strains could therefore induce anti-viral resistance and could protect the enterocytes. Ovine IFN-ω cDNA was cloned into pCYT and pSEC plasmids for intracellular (pCYT:IFN) and secreted (pSEC:IFN) production respectively [5]. Induction of recombinant NZ(pCYT:IFN) and NZ(pSEC:IFN) strains were performed at equal level and IFN-ω production was measured. The levels of IFN-ω activity showed that i) an active form of IFN-ω was produced in both strains, and ii) the activity of IFN-ω found in the supernatant and cell fractions of NZ(pSEC:IFN) strain was about two-fold higher than that observed for the cytoplasmic form (Table 2). Similarly to what was observed for Nuc and E7, secretion leads to higher heterologous protein yields.

## Better secretion for better yields

*L. lactis *has been engineered to secrete of a wide variety of heterologous proteins from bacterial, viral or eukaryotic origins (Table 1). There are reports about secretion bottlenecks and biotechnological tools for heterologous secretion in model bacteria such as *Escherichia coli *and *Bacillus subtilis *[23,24], but only few data are available concerning this aspect in *L. lactis*. Protein size, nature of the SP and presence of a propeptide are parameters that may interfere with protein secretion. Some data available about these features are compiled here.

To optimize secretion and thus production yields, the nature of the SP was the first parameter to modify on heterologous precursor as previously shown using Nuc as a reporter protein. The replacement of the native staphylococcal SP_Nuc _by the homologous lactococcal SP_Usp45 _to direct the secretion of Nuc in *L. lactis *led to an increased SE [25] (Table 3). On the other hand, the replacement of SP_Nuc _by SP_Usp45 _did not enhance the SE of NucT (a truncated mature moiety of Nuc devoid of N-terminal propeptide) suggesting the importance of the propeptide in the SE for Nuc [25] (Table 3). However, in several cases, the use of a homologous SP (and especially SP_Usp45_) allows a better SE compared to a heterologous one. Screening vectors were thus developed to search for new homologous secretion signals in *L. lactis *[1,2]. These screening works offer now a panel of SPs that are suitable for heterologous secretion. However, when compared to SP_Usp45, _the newly described SPs were less efficient to direct secretion of Nuc [1]. Even after a direct mutagenesis on SP310, one of these new SPs identified using a screening strategy [1], the enhanced SE was still lower than the one measured with SP_Usp45 _[26]. However, a recent study by Lindholm *et al. *showed that a *Lactobacillus brevis *SP (originated from a S-layer protein) drove the secretion of the *E. coli *FedF adhesin more efficiently than SP_Usp45 _[27]. High SE might thus result, at least in part, from good adequacy between the mature protein and the SP used to direct secretion.

**Table 3:** Effect of the signal peptide and of the insertion of the LEISSTCDA synthetic propeptide on the secretion efficiency.

**Protein**	**SE^a ^with SP_Nuc_**	**SE with SP_Usp45_**	**Reference**
Nuc	60 %	>95 %	[25]
NucT	30 %	30 %	[25]
**Protein**	**SE without LEISS**	**SE with LEISS**	**Reference**

Nuc	60 %	80 %	[18]
NucT	30 %	90 %	[25]
L7/L12	35 %	50 %	[19]
AmyS^b^	+	+++	[18]

^a^: SE, secretion efficiency is the proportion of total protein which is present in the mature secreted form.

^b^: SE was not determined by western blot and immuno revelation and thus could not be quantified but the activity plate assay demonstrated a clear secretion enhancement (+ to +++) with LEISS.

The fusion of a short synthetic propeptide between the SP and the mature moiety is another innovative biotechnological tool to enhance protein secretion. One such propeptide (composed of nine amino acid residues, LEISSTCDA) was developed and was shown to enhance the SE of several heterologous proteins in *L. lactis*: NucB, NucT, (Table 3) [18], the *B. abortus *L7/L12 antigen (Table 3) [19], and the α-amylase of *Geobacillus stearothermophilus *(Table 3) [18]. Directed mutagenesis experiments demonstrated that the positive effect of LEISSTCDA on protein secretion was due to the insertion of negatively charged residues in the N-terminus of the mature moiety [25]. Furthermore, the enhancement effect does not depend on the nature of the SP, since the secretion of NucB fused to either SP_Nuc _or SP_Usp45 _was enhanced by LEISSTCDA insertion [25]. Strikingly, the enhancement of SE was reproducibly accompanied by an overall increase of protein yields as determined in Western blot experiments. This observation suggests that heterologous precursors are degraded by intracellular proteases when they are not efficiently secreted and that a higher secretion could be a way to escape proteolysis.

## Protein conformation rather than protein size can impair the heterologous protein secretion in *L. lactis*

Proteins with molecular mass ranging from 165 kDa (size of DsrD, the *Leuconostoc mesenteroides *dextransucrase, [28]) to 9.8 kDa (size of Afp1, a *Streptomyces tendae *anti-fungal protein; Freitas et al., submitted) have been successfully secreted in *L. lactis*. This suggests that protein size is not a serious bottleneck for heterologous protein secretion in *L. lactis*. In contrast to protein size, conformation may be a major problem for heterologous secretion in *L. lactis *as illustrated by some recent examples. The first example is the production of the non-structural protein 4 (NSP4) of the bovine rotavirus, the major etiologic agent of severe diarrhea in young cattle. In order to develop live vaccines against this virus, the NSP4 antigen was successfully produced in *L. lactis *[29]. Derivatives of pCYT and pSEC plasmids were constructed to target NSP4 into cytoplasmic or extracellular location. The highest level of production was obtained with the secreted form. However, no secreted NSP4 was detected in the supernatant and both SP_Usp45_-NSP4 precursor and NSP4 mature protein were detected in the cell fraction. Two degradation products were detected in addition to the NSP4 precursor and mature protein. These results suggest that the cytoplasmic form of NSP4 was probably totally degraded inside the cell whereas fusion to the SP_Usp45 _protected NSP4 protein against intracellular proteolysis.

Similar results were obtained when pCYT and pSEC vectors were used to produce the *B. abortus *GroEL chaperone protein: only pSEC:GroEL plasmid was obtained and subsequently the fusion SP_Usp45_:GroEL was detected in Western blot experiments (V. Azevedo, unpublished data). In this case, *B. abortus *GroEL is likely to interact with lactococcal cytoplasmic proteins leading to severe cellular defects and thus to a lethal phenotype. On the other hand, fusion of SP_Usp _to GroEL might keep the chimeric protein in an unfolded and/or inactive state allowing thus its heterologous production.

Another example is the production of the bovine β-lactoglobulin (BLG) in *L. lactis *[30,31]. BLG, a 162 amino acid residues globular protein, is the dominant allergen in cow's milk and was produced in *L. lactis *to test the immunomodulation of the allergenic response in mice when BLG is delivered by a bacterial vector [30]. Western blot and ELISA showed that BLG production was significantly higher when BLG was fused to SP_Usp45 _although the SE was very low, with no detectable BLG in the supernatant of pSEC:BLG strains [30]. Further studies revealed that a fusion between the LEISS propeptide and BLG could not enhance the SE of BLG above ~5%, as determined by ELISA [31].

For rotavirus NSP4, *B. abortus *GroEL, and BLG (which are medium-sized compared to DsrD or Afp1), either very low secretion yields or absence of secretion was observed in *L. lactis*. In all cases, fusion to a SP stabilizes heterologous protein production even though they are not efficiently secreted. These results could be due either to the SP itself that reportedly acts as an intramolecular chaperone or to the protection of the chimeric precursor from intracellular proteolysis by the cytoplasmic chaperones of the Sec-machinery. GroEL (a cytoplasmic chaperone), NSP4 (a structural protein), and BLG (a globular protein) have dramatically different primary sequences. A higher affinity of intracellular housekeeping proteases for these particular sequences cannot be hypothesized since the fusion of a SP leads to the stabilization of the protein. Change of conformation is therefore the predominant criterion involved in the stabilization of the precursors and the higher yields observed. On the other hand, these proteins might undergo rapid folding right after their synthesis, which interferes with (or hampers) the secretion process. Such interferences between protein conformation and SE were previously shown in *E. coli *and *B. subtilis *[32,33]. Altogether, these results suggest that protein conformation rather than protein size is a major problem for heterologous protein secretion in *L. lactis *as well.

## A labile protein can be stabilized by fusion to a stable protein

**Figure 2:**
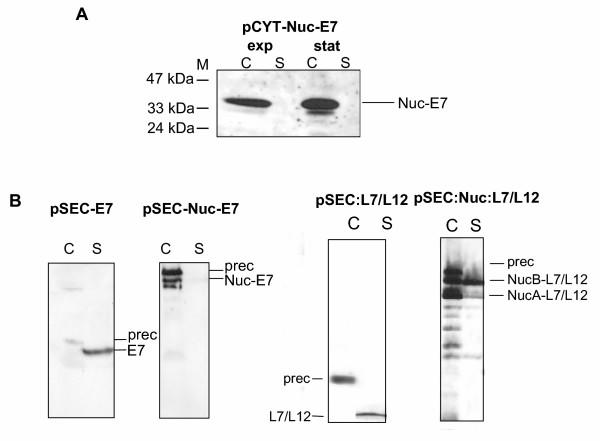
**Fusion to Nuc rescue E7 in intracellular production and increase protein yields for the secreted forms of E7 and L7/L12. A. **A DNA fragment encoding the mature moiety of Nuc was fused to the fragment encoding E7 (pCYT:Nuc:E7). Production of Nuc-E7 analyzed by Western blot using anti-E7 antibodies on protein samples prepared from induced cultures harvested either at exponential (exp) or stationary (stat) phase. Positions and sizes of molecular weight marker (M) are indicated at left. **B. **The mature Nuc fragment was inserted between SP_Usp45 _and the fragment encoding E7 (pSEC:Nuc:E7) or L7/L12 (pSEC:Nuc:L7/L12). Secretion of the fusion proteins was analyzed by Western blot using either anti-E7 or anti-L7/L12 antibodies. C, cell lysates; S, supernatant fraction. Positions of precursor (prec) or mature forms of E7, Nuc-E7, L7/L12, NucB-L7/L12, and NucA-L7/L12 are indicated by arrows.

**Figure 3:**
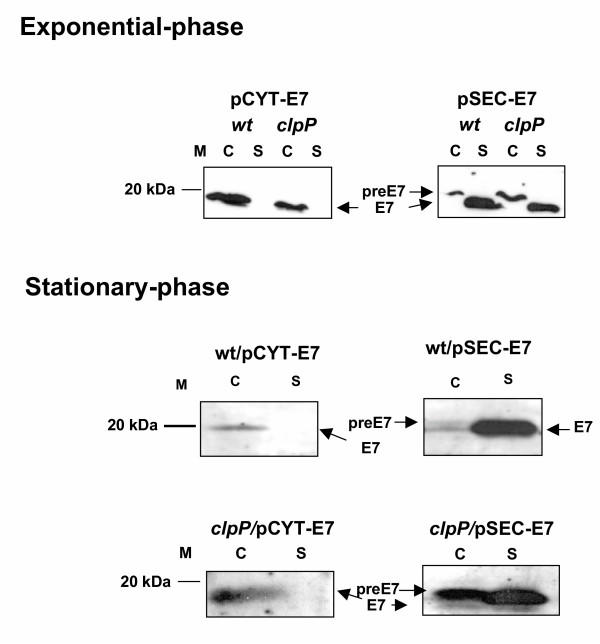
**Native E7 production in wt *L. lactis *depends on growth phase. **E7 production and secretion were analyzed by Western blot from cultures induced at different times so that, 1 hour after nisin induction, the samples are harvested at exponential (OD_600 _= 0.5–0.6, upper panels) or stationary phase (OD_600 _= 1.5, lower panels). wt/pCYT-E7, NZ(pCYT-E7) strain (encoding native E7, cytoplasmic form). wt/pSEC-E7 NZ(pSEC-E7) strain (encoding the precursor preE7). Positions of E7 mature and precursor forms are given by arrows. C, cell lysates; S, supernatant fraction. **ClpP is not involved in the intracellular degradation of E7 in *L. lactis***. Analysis by western blot shows that a strain of *L. lactis *deficient in the intracellular protease ClpP cannot rescue cytoplasmic E7 production. Induced cultures samples of *wt L. lactis *or *L. lactis clpP *mutant strain containing pCYT-E7 (*clpP*/pCYT-E7) or pSEC-E7 (*clpP*/pSEC-E7) taken at exponential- (upper panel) or stationary- (lower panel) phase.

It was clearly demonstrated that the secreted form of E7, a reportedly labile protein, can be stabilized by fusion to Nuc [20,34]. Nuc is reportedly a stable protein and its use, as a fusion partner, does not affect its enzymatic activity. The production of the resulting chimerical protein is thus easy to follow. The cytoplasmic form of E7 was stabilized by the fusion to Nuc even when the production was induced in stationary phase (Fig. 2A), whereas cytoplasmic E7 alone was degraded (see below; Fig. 3). Thus, fusion to the stable Nuc could rescue E7 production in *L. lactis *and allowed higher protein yields compared to E7 alone [20]. Stabilization by fusion to Nuc was observed for several secreted proteins as well. First, a Nuc-E7 fusion on a pSEC backbone resulted in higher production yield although the SE was altered (Fig. 2B). Fusion to the synthetic propeptide LEISSTCDA in a pSEC:LEISS:Nuc:E7 construction restored an efficient secretion yield [34]. Second, in an attempt to increase the protein yield of the secreted L7/L12, a fusion to Nuc (pSEC:Nuc:L7/L12) resulted in a 2.5-fold increase in production yield (Fig. 2B) [19]. Recent results concerning the production of BLG provide a third example of yield enhancement by fusion to Nuc. A pSEC:Nuc:BLG construction allowed a 2-fold increase in BLG yields compared to pSEC:BLG [31]. These results show that Nuc is a stable carrier protein and has a protective effect on labile heterologous chimerical proteins by reducing its sensitivity to intracellular proteolysis. To our knowledge, Nuc is the fusion partner most commonly tested so far for stabilization in *L. lactis*. Bernasconi et al (2002) fused the *Lactobacillus bulgaricus *proteinase PrtB to BLG, which was subsequently stabilized by the PrtB carrier [13]. It is thus difficult to postulate any rule concerning the stabilization effect. Different results (i.e. no stabilization) could perhaps be observed with a different partner and thus could help to determine the mechanism of the stabilization effect. In biotechnological use of recombinant *L. lactis *strains for protein production, fusions can also facilitate purification (e.g. His-tag strategy). Protein fusion has also been successfully used to optimize the production of the two subunits of heterodimeric complexes as demonstrated with murine interleukin-12 in *L. lactis *[22] or with heterodimeric enzymes in *E. coli *[35]. In both cases, the resulting fusion had the expected properties. In other cases however, such fusions might dramatically interfere with the conformation of one or both of the proteins, which might be deleterious for the expected activity. Nevertheless, when *L. lactis *is used as an antigen delivery vector, fusions can be envisioned since it was demonstrated that both moieties of the chimerical protein are still recognized by the corresponding antiserum [10,20,34] and are immunogenic [36].

## Secretion avoids proteolysis?

Several of the results mentioned above suggest that secretion could be an efficient way to escape intracellular proteolysis. This hypothesis was particularly tested in E7 production [20]. E7 was indeed degraded when intracellular production was induced in late exponential or early stationary growth phase (Fig. 3). E7 production was then tested in a *clpP *deficient strain (ClpP is reportedly the major house keeping protease in *L. lactis*; [37]) and in a *dnaK *deficient strain (DnaK is an intracellular chaperone that may promote proteolysis by maintaining the protein in an unfolded state; [38]). In exponential or stationary phase cultures, no significant difference in E7 patterns was observed between wild type and *clpP*^- ^(Fig. 3) or *dnaK*^- ^(not shown) strains: E7 was equally degraded in the cytoplasm and remained unchanged in supernatants samples. Altogether, these results indicate that E7 intracellular proteolysis is ClpP- and DnaK- independent. Until recently, only two cytoplasmic proteases, ClpP and FtsH [39], have been identified in *L. lactis*. The existence of a third, as yet unidentified protease was postulated by studies of a *clpP *mutant suppressor [40]. E7 may thus be a useful screening target to identify a putative *L. lactis *protease that, as suggested by our data, is activated in stationary phase.

**Figure 4:**
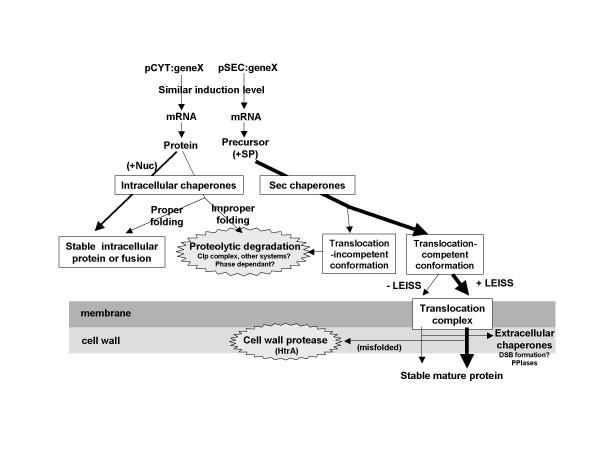
**Schematic presentation of the molecular tools and the cellular events that can affect the production yields of heterologous protein in *L. lactis***. Thicknesses of the arrows are proportional to the final production yields. All the host factors involved in the cellular events are not identified and or characterized yet. SP, signal peptide (encoded in pSEC constructions), +Nuc, fusion between the protein of interest and the stable Nuc protein.

Besides the features of the precursor itself, these results also rise that host factors are involved in protein stability and SE (Fig. 4). Research efforts are now focusing on the analysis of host factors involved in protein production and secretion by either directed or random mutagenesis in *L. lactis *[41].

Although *L. lactis *possesses a wide range of enzymes (peptidases, housekeeping proteases) dedicated to intracellular proteolysis, it possesses only one extracellular housekeeping protease (HtrA) [9] and its major extracellular scavenger protease, PrtP, is plasmid encoded [42]. Thus, a plasmidless strain does not present any protease activity in the medium. Better production yields could then be expected when secretion is used *versus *cytoplasmic production. These results give clues and provide the research workers with target proteins to study intracellular proteolysis and protein stability inside and outside the host strain. Such studies already led to the development of *htrA *deficient *L. lactis *strains. Heterologous protein secretion and anchoring in a *htrA *deficient strain allowed higher protein stability at the cell surface for several heterologous proteins [10].

## Perspectives

Current research works are now focusing on other host factors that affect protein production and secretion in *L. lactis*. *L. lactis *complete genome sequence analysis revealed indeed that the Sec machinery comprises fewer components than the well-characterized *B. subtilis *Sec machinery. Notably, *L. lactis *does not possess any SecDF equivalent and complementation of the lactococcal Sec machinery with *B. subtilis *SecDF results in better secretion yields as determined for Nuc reporter protein (Nouaille et al., submitted). Random mutagenesis approaches also revealed that features of some cell compartment, such as the cell wall, play an important role in the secretion process [41]. Similar approaches allowed the identification and characterization of genes of unknown functions specifically involved in production yields of the secreted proteins in *L. lactis *(Nouaille et al., in preparation).

Many molecular tools are now available to direct heterologous protein secretion in *L. lactis *and the list of heterologous proteins produced in this bacterium is regularly increased. The reports where cytoplasmic and secretion production can be compared mostly show that secretion allows better protein yields compared to intracellular production; and allow a better understanding of the protein production and secretion process in *L. lactis*.

Future works should investigate the *L. lactis *capacities for protein modifications. For example, we showed that proteins that require a disulfide bond (DSB) to acquire their native conformation can be efficiently produced and secreted in *L. lactis *[5,22,27]. However, no equivalent of *E. coli dsb *or *B. subtilis bdb*, the genes involved in DSB formation, was found by sequence comparison in *L. lactis*. Similarly, other folding elements (i.e. PPIases, so-called maturases...) are still to be identified and the *L. lactis *capacities for post-translational modifications are still to be investigated.

Altogether, these works will contribute to the development and the improvement of new food-grade systems for *L. lactis *[43] and should lead, in a near future, to the construction of lactococcal strains dedicated to high-level production of proteins of interest. The GRAS status of *L. lactis *and LAB in general, is a clear advantage for their use in production and secretion of therapeutic or vaccinal proteins.
